# Where to Send Christmas Boxes

**Published:** 1892-12-24

**Authors:** 


### Where to Send Christmas Boxes.

We are constantly asked by those charitable people who think before they act, what charities really deserve to be helped, and how the Christmas gifts of the benevolent may be put to the best possible use. With the view of helping those persons to satisfy both their judgment and their generous instincts at the same time, we give here the briefest possible summary of the work of the hospitals during a single year. It is quite certain that money given to bona fide hospitals is wisely and well spent. In the first place, the hospitals expended in 1891 the sum of £586,172, and they have spent something like that sum annually for several years. This amount is not all raised by *de novo* efforts every year. There are certain capitalised resources to draw upon and other sources of regular income. Let us be thankful that it is so. The existence of these resources brings the case of the sick well within the reach of every year's power of giving, if only everybody would give what he could. The amount of income that can be depended upon, whether outside people give much or little, is £344,500. This is a very respectable sum as a basis of operations. But in order to meet the expenditure that is actually made, and of which the sum of £586,172 ought to represent the minimum, as already stated, it is necessary that each year should raise within its own twelve months the very considerable further sum of £241,622. This for London alone. The task looks great, and it is great. But then the power which accomplishes it is also great. It is less than one sovereign for every fifty of the population, or less than sixpence for every individual. Looked at in this way the task compared with the money power is by no means gigantic. Consider for a moment the bare outline of the work which this large sum produces? The general hospitals, of which there are 23, treat 51,361 in-patients and 549,310 out-patients annually. There are 5 chest hospitals, and these receive, of in-patients 4,611, and of out-patients 40,826. Surely these hospitals for diseases of the chest appeal to thousands and tens of thousands of hearts who know by painful experience the deadly doings of London's winters and London's fogs. The children's hospitals number 11, and they treat 6,000 in-patients and 91,059 out-patients. Would anybody diminish these children's hospitals by a single bed, or turn one suffering child helpless away because the last bed was filled and the last funds were exhausted? There are 10 hospitals for women, including lying-in hospitals, and these treat 4,822 in-patients and 34,828 out patients. Other special hospitals, which include those for the Eye, for the Ear and Throat, the Cancer Hospital, hospitals for the Paralysed, for Epileptics, for diseases of the Skin, Orthopœdic hospitals, &c., treat together 7,417 in-patients and 155,911 out-patients. Beside these there are convalescent homes, cottage hospitals, and various sick institutions, which bring up the grand total, as we have already stated, to 88,562 in-patients and 874,048 out-patients, at an annual cost of £586,172, of which £241,622 is required to be raised annually in donations, subscriptions, and other forms of aid. These figures, when rightly understood, appeal with irresistible power to every kindly heart.

